# Acute Dehydration Drives Organ-Specific Modulation of Phosphorylated AQP4ex in Brain and Kidney

**DOI:** 10.3390/ijms27020617

**Published:** 2026-01-07

**Authors:** Claudia Palazzo, Roberta Pati, Raffaella Pia Gatta, Onofrio Valente, Pasqua Abbrescia, Grazia Paola Nicchia, Antonio Frigeri

**Affiliations:** 1 Department of Translational Biomedicine and Neurosciences, University of Bari Aldo Moro, 70124 Bari, Italy; claudia.palazzo@uniba.it (C.P.); r.gatta3@phd.uniba.it (R.P.G.); onofrio.valente@uniba.it (O.V.); pasqua.abbrescia@uniba.it (P.A.); 2 Department of Bioscience, Biotechnology and Environment, University of Bari Aldo Moro, 70126 Bari, Italy; roberta.pati@uniba.it (R.P.); graziapaola.nicchia@uniba.it (G.P.N.)

**Keywords:** Aquaporin-4, dehydration, homeostasis

## Abstract

Water deprivation triggers coordinated physiological responses to preserve body fluid balance, yet the molecular mechanisms that regulate aquaporin-mediated water transport under dehydration remain incompletely understood. Aquaporin-4 (AQP4), the main water channel in the brain and a basolateral water pathway in the kidney collecting duct, exists in multiple isoforms, including the translational readthrough variant AQP4ex, whose regulatory role is only beginning to be defined. Here, we investigated the effects of acute water deprivation (6–12 h) on AQP4 isoform expression and phosphorylation in a mouse kidney and brain. While total AQP4 and AQP4ex protein levels remained largely unchanged in both tissues, dehydration induced a marked and divergent regulation of the phosphorylated form of AQP4ex. Levels increased in the kidney medulla, consistent with enhanced antidiuretic water transport, but decreased in the cerebral cortex, suggesting a protective reduction in perivascular water permeability. No changes were detected in the cerebellum. These findings identify phosphorylation of AQP4ex as a rapid, tissue-specific regulatory mechanism that adjusts water flux according to the physiological needs of each organ, revealing an additional layer of control in systemic water homeostasis and highlighting AQP4ex as a potential target in dehydration-related and osmotic disorders. Future studies could explore the signaling pathways regulating AQP4ex phosphorylation and investigate its potential involvement in pathological conditions, such as diabetes insipidus or cerebral edema.

## 1. Introduction

Water homeostasis is a fundamental requirement for the normal functioning of organisms, supporting cellular volume regulation, interstitial fluid balance, and overall osmotic stability. The aquaporin channel family (AQPs) provides regulated pathways for bidirectional water movement across cell membranes in response to osmotic and hydrostatic gradients [1,2,3], thereby playing central roles in both steady-state fluid balance and adaptive responses to water stress [4,5]. Among aquaporins, Aquaporin-4 (AQP4) receives particular attention because of its distinctive tissue distribution, isoform diversity, and involvement in both physiological and pathophysiological processes.

Aquaporin-4 (AQP4) is widely expressed, with the highest densities in astrocyte endfeet at the blood–brain barrier, where it regulates bidirectional water flux and contributes to the maintenance of fluid homeostasis [6]. Structurally, each AQP4 monomer contains six transmembrane helices forming a narrow pore of ~2.8 Å, and four monomers that assemble into tetramers [7]. Tetramers further assemble into higher-order supramolecular structures known as orthogonal arrays of particles (OAPs) [8], which are observable by freeze-fracture electron microscopy [9]. The functional regulation of AQP4 depends not only on its overall expression level but also on isoform composition, supramolecular organization, subcellular localization, phosphorylation state, and interactions with membrane-associated scaffolding proteins [8].

Two canonical AQP4 isoforms, M1 and M23, are translated from distinct start sites of the same transcript and differ in their N-terminal sequences [8]. The shorter M23 isoform strongly promotes OAPs formation, whereas the longer M1 isoform preferentially forms tetramers with reduced ability to assemble into large arrays [10]. The relative abundance of these isoforms is proposed to influence water permeability, lateral mobility within the membrane, and anchoring to cytoskeletal or scaffolding proteins [8]. More recently, “extended” AQP4 isoforms (M1ex and M23ex), generated by translational readthrough, have been identified and characterized [11]. This extended C-terminus confers unique regulatory features to AQP4ex, including specific post-translational modifications such as phosphorylation [12] and a preferential localization at perivascular astrocytic endfeet [13,14]. Importantly, functional studies in AQP4ex-deficient mouse models have demonstrated that the extended isoforms retain water transport properties comparable to those of the canonical AQP4 isoforms [13], indicating that the C-terminal extension does not impair channel permeability but rather modulates AQP4 localization, anchoring, and regulation. The growing recognition of isoform complexity underscores the need to understand how each variant contributes to fluid regulation at both cellular and tissue levels.

In water regulation and homeostasis, AQP4 isoforms play several key roles. In the central nervous system, the polarized distribution of AQP4 at astrocytic endfeet facilitates efficient water exchange between the perivascular space, interstitial fluid, and cerebrospinal fluid, supporting the glymphatic clearance system [15,16]. In the kidney, AQP4 is expressed in the basolateral membrane of principal cells in the collecting duct, where it provides a constitutive pathway for water to exit into the interstitium. Although AQP4 knockout alone results in only mild urinary concentrating defects, its role in fine-tuning water flux remains physiologically relevant [17,18,19].

Under dehydration or hypo-hydration stress, water regulatory mechanisms must shift from the maintenance of equilibrium toward the preservation of intracellular and extracellular volume and the prevention of osmotic imbalance. Renal responses to water deprivation include up-regulation of the vasopressin-sensitive water channel AQP2 in the collecting duct, along with coordinated regulation of other aquaporins such as AQP3 and AQP4 [20,21]. For example, in chickens subjected to water deprivation for 48 h, AQP4 mRNA increases in the hypothalamus but decreases in the kidney, indicating tissue-specific regulation under osmotic challenge [20]. In mammals, studies in rats show that brain AQP4 mRNA increases during water deprivation [22,23]. Together, these findings indicate that AQP4 isoforms and their regulation are integral to dehydration responses in both the CNS and peripheral tissues.

Although significant progress has been made, we still do not fully understand how the changes in AQP4 isoforms expression, the aggregation in OAPs, and phosphorylation events could work together to help the whole organism conserve water during dehydration.

While isoform composition has been linked to OAPs formation and water permeability [10], direct in vivo studies connecting isoform dynamics to dehydration outcomes are still limited. Moreover, the roles of extended isoforms (M1ex/M23ex) in peripheral epithelia during dehydration remain largely unexplored. In this context, the present study examines the expression levels and localization of AQP4 isoforms in the brain and kidney, the two tissues with the highest physiological expression of AQP4, during controlled acute water deprivation. Our results demonstrate that AQP4ex exhibits a dynamic and tissue-specific regulatory pattern, including changes in its phosphorylated form.

## 2. Results

### 2.1. Effect of Acute Water Deprivation on Urine and Plasma Osmolality

**Figure 1:**
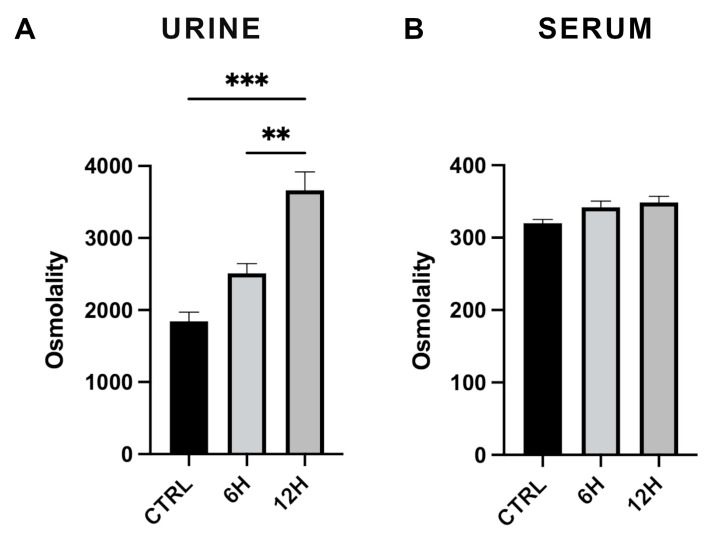
Urine and serum osmolality in mice after water deprivation. (**A**) Urine osmolality measured in control mice (CTRL) and in mice subjected to 6 or 12 h of water deprivation. Urine osmolality increased markedly and in a time-dependent manner during dehydration. (**B**) Serum osmolality showed a slight increase after 6 and 12 h of water deprivation, while remaining within the physiological range. Data are presented as mean ± SEM. Statistical analysis was performed using one-way ANOVA (** *p* < 0.01; *** *p* < 0.001; n = 4 per group).

Urine and plasma osmolality were measured in mice subjected to acute water deprivation for 6 or 12 h and compared with normally hydrated controls. As expected, a time-dependent increase in urinary osmolality was observed: values were already elevated at 6 h and further increased at 12 h (Figure 1), indicating progressive activation of the kidney’s concentrating mechanisms in response to dehydration. Plasma osmolality also showed an upward trend, although the changes did not reach statistical significance compared to controls.

Thus, in mice, acute water deprivation elicits a robust and statistically significant increase in urinary osmolality, reflecting activation of the vasopressin-dependent concentrating mechanism. In parallel, plasma osmolality displays a detectable, albeit non-significant, increase that is nonetheless sufficient to trigger central osmosensory pathways [24].

### 2.2. AQP4 Isoforms Expression and Localization in Mouse Kidney After Water Deprivation

**Figure 2:**
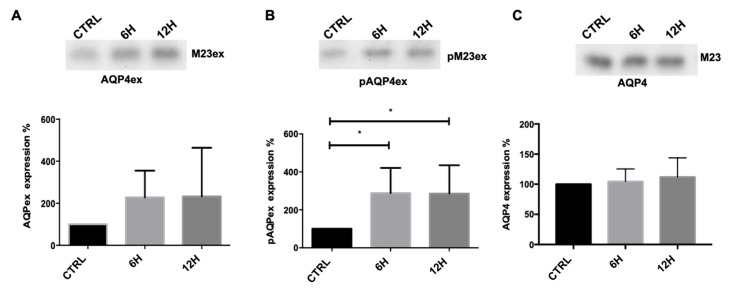
Immunoblot analysis of AQP4 isoforms in mouse kidney medulla after water deprivation. Top, representative immunoblot of AQP4 isoforms in kidney medulla derived from control mice (CTRL) and mice deprived of water for 6 and 12 h were incubated for 6 and 12 h, incubated with antiAQP4ex (**A**), anti-pAQP4ex (**B**), and anti-AQP4 (**C**) antibodies. Bottom, bar graphs showing the mean ± SEM of protein expression levels, expressed as percentage of CTRL. One-way ANOVA, * *p* < 0.05, n = 7.

To investigate the regulation of AQP4 and its extended isoforms (AQP4ex) under water deprivation, expression levels were first analyzed by immunoblotting (Figure 2) using a membrane vesicle fraction prepared from the kidney medulla, which enriches membrane-associated proteins and increases detection sensitivity compared with total tissue homogenates. Immunoblot detection and densitometric analysis showed an increase in AQP4ex isoforms, particularly in the phosphorylated form (pAQP4ex), in both the 6 h and 12 h dehydration groups (Figure 2A,B). Interestingly, despite this increase, total AQP4 protein levels remained stable across all conditions (Figure 2C), suggesting a post-transcriptional or isoform-specific regulatory mechanism rather than a global induction of AQP4.

These findings indicate that water deprivation modulates the expression of AQP4ex in the kidney, with p-AQP4ex showing a particularly marked and statistically significant increase, potentially contributing to the adaptive modulation of water permeability in the collecting duct under antidiuretic stress.

Immunofluorescence experiments were performed to assess whether the spatial distribution of AQP4ex in the kidney is altered following water restriction.

**Figure 3:**
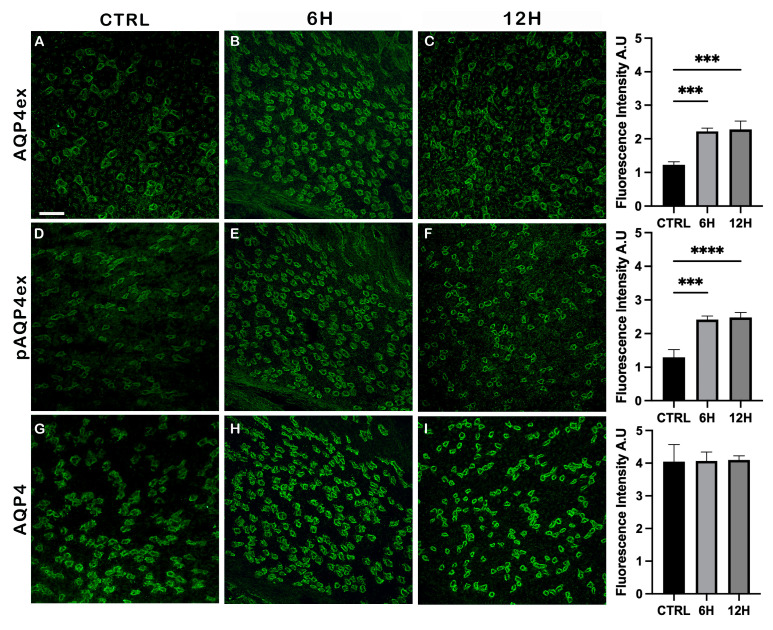
Immunofluorescence analysis of AQP4 isoforms in mouse kidney after water deprivation. Strong staining of AQP4ex (**A**–**C**), pAQP4ex (**D**–**F**), and total AQP4 (**G**–**I**) is observed at the basolateral membrane of the principal cells in the collecting duct. Scale bar: 75 μm. Bar graphs showing the quantification of AQP4 isoforms fluorescence intensity (mean ± SEM), in control mice (CTRL) and after 6 h and 12 h of water deprivation. One-way ANOVA *** *p* < 0.001, **** *p* < 0.0001.

As shown in Figure 3, AQP4ex labeling is mainly restricted to the renal medulla, where it localizes to the basolateral membrane of principal cells in the collecting duct, consistent with previous reports [14]. The pAQP4ex displays a staining pattern that overlaps with AQP4ex and is indistinguishable from that of the canonical AQP4 isoforms.

No changes in the distribution pattern were observed in dehydrated kidneys; however, immunofluorescence analysis revealed a significant increase in AQP4 isoform intensity after water deprivation, in agreement with the elevations detected by immunoblot analysis.

### 2.3. AQP4 Isoforms Expression and Localization in Mouse CNS After Water Deprivation

**Figure 4:**
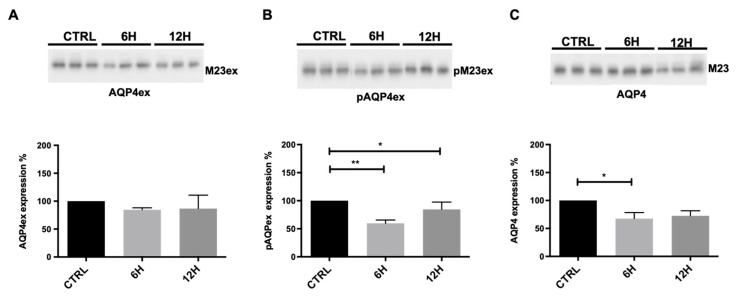
Immunoblot analysis of AQP4 isoforms in mouse brain. Top, representative immunoblot with total lysates, derived from control mice (CTRL) and mice deprived of water for 6 and 12 h, incubated with anti-AQP4ex (**A**), anti-pAQP4ex (**B**), and anti-AQP4 (**C**) antibodies. Bottom, bar graphs showing the mean ± SEM of protein expression levels, expressed as percentage of CTRL. One-way ANOVA * *p* < 0.05, ** *p* < 0.01, n = 7.

To determine whether dehydration modulates AQP4 expression in the CNS, we analyzed AQP4 isoforms in the mouse brain and cerebellum. Immunoblot analysis of total brain cortex homogenates from mice subjected to water deprivation revealed a marked decrease (~50%) in pAQP4ex after 6 h of dehydration compared with controls (Figure 4B). After 12 h of water deprivation, pAQP4ex levels appeared to partially recover but remained significantly lower than in controls (~60–70% of control; Figure 4C). In contrast, total AQP4ex expression levels remained stable across all time points (Figure 4A), indicating that the observed changes reflect post-translational regulation rather than altered protein abundance. Furthermore, total AQP4 protein levels showed a slight reduction after 6 h of water deprivation.

**Figure 5:**
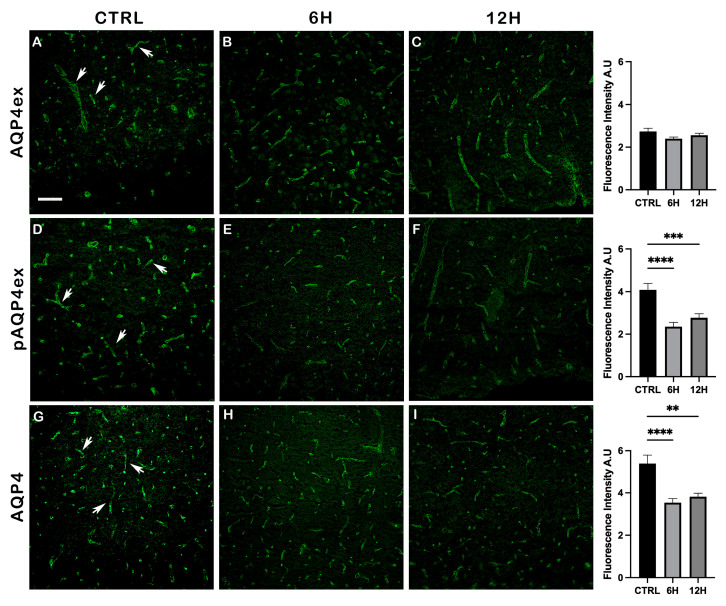
Immunofluorescence analysis of AQP4 isoform expression in mouse brain cryosections after water deprivation. The perivascular staining, highlighted by arrows, is observed in control mice (**A**,**D**,**G**), dehydrated for 6 h (**B**,**E**,**H**), and for 12 h (**C**,**F**,**I**). Scale bar: 75 μm. Bar graphs showing the quantification of AQP4 isoforms fluorescence intensity (mean ± SEM), in control mice (CTRL) and after 6 h and 12 h of water deprivation. One-way ANOVA ** *p* < 0.01, *** *p* < 0.001, **** *p* < 0.0001.

To complement immunoblot findings, an immunofluorescence experiment was performed on the brain cortex (Figure 5).

In control (hydrated) mice, pAQP4ex staining was intense and spatially co-localized with total AQP4ex (Figure 5A–C), consistent with prior observations in the human brain, that pAQP4ex is confined to perivascular astrocyte endfeet overlapping AQP4ex domains [12]. After 6 h of dehydration, qualitative inspection and quantitative analysis revealed a decrease in pAQP4ex at astrocyte endfeet (Figure 5E), whereas the AQP4ex signal remained unchanged and retained its perivascular localization (Figure 5B). At 12 h dehydration, pAQP4ex staining remained significantly weaker than controls (Figure 5F), and without apparent alteration in AQP4ex (Figure 5C). Canonical AQP4 appeared not affected in localization and signal intensity in all experimental conditions (Figure 5G–I).

This immunofluorescence analysis corroborates the immunoblot results, demonstrating that dehydration primarily affects the phosphorylation state of the extended isoform.

Next, we assessed the impact of water deprivation in the mouse cerebellum, where AQP4 is strongly expressed in the astrocyte network of the granular cell layer (gcl). In this region, AQP4 also displays a diffuse, non-perivascular distribution that is independent of dystrophin expression

**Figure 6:**
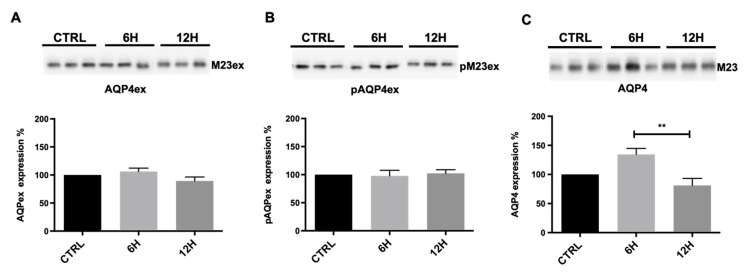
Immunoblot analysis of AQP4 isoforms in mouse cerebellum. Top, representative immunoblot with total lysates, derived from control mice (CTRL) and mice deprived of water for 6 and 12 h, were incubated with anti-AQP4ex (**A**), anti-pAQP4ex (**B**), and anti-AQP4 (**C**) antibodies. Bottom, bar graphs showing the mean ± SEM of protein expression levels, expressed as percentage of CTRL. One-way ANOVA ** *p* < 0.01; n = 7.

Neither AQP4 nor AQP4ex and pAQP4ex levels showed significant differences after 6 h or 12 h of dehydration when compared to controls (Figure 6), indicating that their expression remained stable under dehydration in the cerebellum.

**Figure 7:**
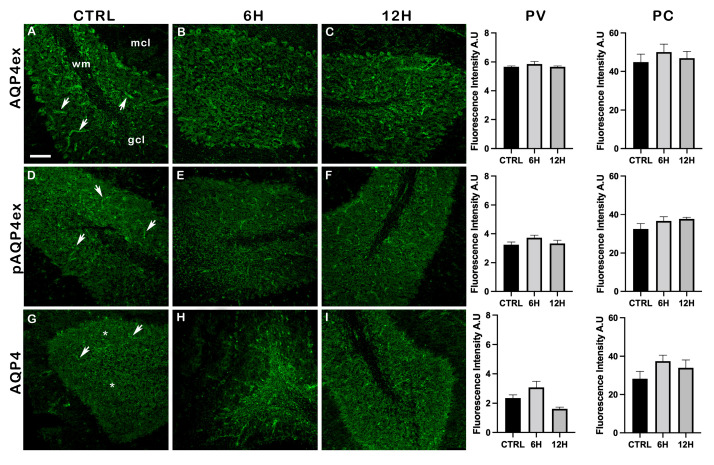
Immunofluorescence analysis of AQP4 isoforms in mouse cerebellum after water deprivation. The molecular cell layer (mcl), the granular cell layer (gcl), and the white substance (wm) are highlighted in panel A. The AQP4ex perivascular staining, highlighted by arrows, is observed in control mice (**A**,**D**), dehydrated for 6 h (**B**,**E**), and for 12 h (**C**,**F**). The diffuse parenchymal signal from AQP4, marked by asterisks (**G**), is shown in control mice (**G**) and in mice deprived of water for 6 h (**H**) and 12 h (**I**). Scale bar: 75 μm. Bar graphs showing the quantification of AQP4 isoforms fluorescence intensity (mean ± SEM), in control mice (CTRL) and after 6 h and 12 h of water deprivation. Fluorescence analysis was performed separately for perivascular (PV) and parenchymal (PC) AQP4 signals. One-way ANOVA.

Confocal imaging of cerebellar sections of dehydrated mice stained with AQP4 antibodies is shown in Figure 7. AQP4ex and pAQP4ex labeling was strong and mainly localized perivascularly within the gcl, while the expression of the canonical isoforms is also non-perivascular [17]. The staining intensity of both extended and canonical AQP4 appeared qualitatively and quantitatively unchanged in dehydrated mice at both 6 h and 12 h compared to controls (Figure 7). These data suggest that, in the cerebellum, unlike the brain, water deprivation does not significantly affect AQP4 isoforms.

## 3. Discussion

The present study demonstrates that acute water deprivation induces selective, tissue-specific regulation of the phosphorylated form of AQP4ex, whereas total AQP4 and AQP4ex protein levels remain largely unchanged. This indicates that post-translational modification, rather than altered protein abundance, represents the primary mechanism by which AQP4ex responds to osmotic stress. Notably, the kidney and brain display opposite regulatory patterns, suggesting distinct physiological roles for AQP4ex depending on the organ’s functional priority during dehydration.

### 3.1. Renal Response: Phosphorylation of AQP4ex Supports Water Reabsorption

In the kidney medulla, dehydration caused a marked increase in pAQP4ex in the collecting duct, coinciding with the rise in urine osmolality, a classical hallmark of vasopressin-dependent antidiuretic activation [24]. Since basolateral AQP4 mediates water efflux from the principal cell after vasopressin-regulated AQP2 insertion at the apical membrane [25,26], enhanced phosphorylation of AQP4ex may contribute to channel stabilization or membrane retention during maximal water recovery. Unlike AQP2, which is mainly regulated at the transcriptional and trafficking level via cAMP/PKA signaling [26,27,28], AQP4 is thought to be modulated, at least in part, through phosphorylation-dependent processes [29,30]. The selective targeting of the AQP4ex C-terminal extension may represent an additional layer of modulation that could complement vasopressin-mediated control of water balance.

### 3.2. Cerebral Response: Reduced pAQP4ex as a Protective Mechanism

In contrast, dehydration in the cerebral cortex led to a significant decrease in pAQP4ex, whereas total AQP4 levels and localization remained unchanged. Immunofluorescence confirmed a selective reduction in the phosphorylated isoform at astrocytic endfeet, where AQP4ex anchors AQP4 to the dystrophin-associated protein complex [13,31,32]. This down-regulation may serve to restrict transcapillary water movement and preserve astrocyte volume, an essential function in the rigid intracranial environment where changes in osmolality can impair neuronal function [33,34]. Unlike the brain cortex, the cerebellum, where AQP4 is also strongly expressed in non-perivascular astrocytes, displayed no significant modulation of AQP4ex or its phosphorylated form, further indicating that AQP4 regulation is highly region-dependent.

The opposite responses of the kidney and brain may reflect two complementary survival mechanisms: the kidney must maximize water conservation to maintain blood volume [35], whereas the brain must minimize cellular shrinkage and protect ionic homeostasis [36,37]. These observations support the idea that AQP4ex does not function merely as a structural variant of AQP4, but as a regulatory element capable of adapting water permeability to the physiological demands of different tissues.

### 3.3. Role of AQP4ex Phosphorylation

Although AQP4ex represents only about 15% of total AQP4, its C-terminal extension carries additional regulatory residues, including Ser335, which we previously identified as a phosphorylation site in the human brain [12]. Given its evolutionary conservation, this residue likely enables dynamic modulation of AQP4-syntrophin interactions and OAPs organization [38]. Thus, AQP4ex may act as a qualitative regulator of water channel assembly and localization rather than as a major determinant of total permeability.

The specific kinases and phosphatases responsible for Ser335 regulation remain unknown, although PKC- and PKA-dependent phosphorylation of canonical AQP4 has been documented [29,30,39]. Further studies should determine whether modulation of AQP4ex phosphorylation directly affects water transport, OAPs stability, or glymphatic function, and whether the response is reversible upon rehydration or prolonged osmotic stress.

In summary, acute dehydration triggers opposing phosphorylation responses of AQP4ex in the kidney and brain, revealing a previously unrecognized layer of organ-specific water regulation. Phosphorylation of AQP4ex may act as a rapid, reversible mechanism that adjusts water flux according to physiological need: enhancing reabsorption in the kidney while protecting structural integrity in the brain. These findings expand the current understanding of AQP4 biology and identify AQP4ex as a potential therapeutic target in conditions of disrupted water homeostasis, including dehydration, cerebral edema, and neurological disease.

## 4. Materials and Methods

### 4.1. Animals

Experiments were conducted in accordance with the European directive on animal use for research, and the project was approved by the Institutional Committee on Animal Research and Ethics of the University of Bari and the Italian Health Department (protocol code 671/2021-PR, approved on 10 September 2021). Experiments were performed on adult mice. Mice were maintained under a 12 h dark/light cycle, with a constant room temperature and humidity (22  ±  2 °C, 75%), and had access to food and water *ad libitum*. All experiments were designed to minimize the number of animals used and their suffering. To assess the effects of short-term water deprivation, mice were randomly divided into three groups: a control group, which received food and water *ad libitum*; a 6 h water deprivation group; and a 12 h water deprivation group. During the deprivation periods, mice continued to have access to food. All experiments were conducted at the same time of day to minimize circadian variability. At the end of the treatment period, animals were euthanized by approved methods, and tissues were rapidly collected, frozen in liquid nitrogen, and stored at −80 °C until further analysis.

### 4.2. Urine and Serum Chemistry

Blood samples were collected by cardiac puncture. Urine and serum osmolarities were measured using a vapor pressure osmometer (model 5600, Wescor, Logan, UT, USA). Urine samples were diluted 1:10 prior to measurement, whereas serum samples were analyzed undiluted.

### 4.3. Antibodies

The following primary antibodies were used: rabbit polyclonal anti-AQP4 (1 mg/mL, Sigma, Saint Louis, MO, USA) diluted to 1:8000 for immunoblot analysis and 1:2000 for immunofluorescence. A custom anti-pAQP4ex affinity-purified polyclonal antibody was generated by GenScript Biotech (Piscataway, NJ, USA) against the mouse peptide containing the phosphorylated Ser335 (0.359 mg/mL), diluted at 1:2000 for immunoblot and immunofluorescence. Custom rabbit polyclonal anti-mouse AQP4ex (GenScript, Piscataway, NJ, USA) was generated against the peptide DSTEGRRDSLDLASC within the mouse AQP4 carboxy-extension (0.972 mg/mL) and diluted 1:2000 for immunoblotting and 1:2000 for immunofluorescence. For immunofluorescence, AlexaFluor 488 anti-rabbit was used (Life Technologies, Thermo Fisher Scientific, Carlsbad, CA, USA) at a dilution of 1:1000; for immunoblotting, anti-rabbit IgG-HRP (Bio-Rad, Hercules, CA, USA) was used at 1:3000.

### 4.4. Immunofluorescence on Tissue Sections

Immunofluorescence experiments were performed as previously described [40]. Briefly, isolated tissues were fixed in 4% PFA solution at 4 °C overnight and then, after washing for 1 h in PBS, were immersed in 30% sucrose solution in PBS overnight. After washing in PBS, tissues were embedded in Tissue-Tek OCT compound (Sakura Finetek, Alphen aan den Rijn, The Netherlands) and frozen at −80 °C. Tissues were sliced at 10 μm thickness using a cryostat (CM 1900; Leica, Wetzlar, Germany) at −20 °C and collected on SuperFrost Plus adhesion slides (Thermo Fisher Scientific, Waltham, MA, USA).

After blocking, sections were incubated with primary antibodies for 1 h at room temperature in blocking solution (0.1% Gelatin in PBS), washed for 30 min, and then incubated with secondary antibodies for 1 h. Finally, the sections were washed for 15 min in PBS and mounted in PBS-glycerol (1:1) pH 8.0, containing 1% n-propyl gallate.

Finally, sections were viewed with a Leica DM2500 LED fluorescence microscope using 20X/0.55 and 40X/0.80 PL FLUOTAR objectives and photographed with a Leica DFC7000 T CCD camera. Confocal images were obtained using an automated inverted Leica TCS SP8 confocal microscope with a 40X HC PL Apo oil CS2 objective.

### 4.5. Quantitative Immunofluorescence Analysis

Quantitative analysis of fluorescence intensity was performed using Fiji/ImageJ software (version 1.54r, NIH, Bethesda, MD, USA). In kidney sections, fluorescence intensity was quantified by placing circular ROIs of identical size around AQP4 isoforms-positive collecting ducts, identified based on immunoreactivity. Mean fluorescence intensity was measured within each ROI.

To assess perivascular fluorescence in brain sections, linear regions of interest (ROIs) were drawn perpendicularly to cerebral capillaries [41], identified by AQP4 isoform antibodies. Line profiles were extracted using the plot profile function to obtain fluorescence intensity values across the capillary wall and adjacent perivascular regions. For each animal, multiple capillaries were analyzed across independent sections, and the resulting values were averaged.

In cerebellar sections, AQP4 isoforms fluorescence was quantified separately in distinct cellular pools. Perivascular signal was assessed using linear ROIs drawn perpendicular to capillaries, and fluorescence intensity values from multiple line profiles were averaged per vessel. Diffuse parenchymal signal was quantified using circular ROIs of identical size placed in vessel-free regions.

For all tissues, background fluorescence was measured in areas devoid of specific staining and subtracted from all measurements. Multiple ROIs were analyzed across independent sections for each animal.

### 4.6. Plasma Membrane Vesicles from Kidney Medulla

Kidney medullas were excised and homogenized in 5 volumes of ice-cold homogenizing buffer (250 mM Sucrose, 10 mM Tris-HCl, pH 7.5) with an added complete protease inhibitor cocktail (Roche, Milan, Italy) for protein stability. The homogenate was centrifuged at 800× *g* for 10 min at 4 °C, and the supernatant was first centrifuged at 8000× *g* for 10 min and then centrifuged at 17,000× *g* for 45 min at 4 °C to obtain a low-speed pellet enriched in AQP4-containing plasma membrane vesicles.

### 4.7. Sample Preparation for SDS-PAGE

For SDS-PAGE [42], harvested tissues were frozen in liquid nitrogen and stored at − 80 °C. Proteins were extracted in 5–7 volumes of RIPA buffer (10 mM Tris-HCl, pH 7.4; 140 mM NaCl; 1% Triton X-100; 1% Na + deoxycholate; 0.1% SDS; 1 mM Na3VO4; 1 mM NaF and 1 mM EDTA) added with a cocktail of protease inhibitors (Roche, Milan, Italy). The lysis was performed on ice for 30 min, and the samples were then centrifuged at 17,000× *g* for 1 h at 4 °C. The supernatant was collected, and the proteins were measured with a bicinchoninic acid (BCA) Protein Assay Kit (Thermo Scientific, Waltham, MA, USA).

### 4.8. SDS-PAGE and Western Blot Analysis

The electrophoresis and immunoblotting were performed as previously described [40]. Briefly, proteins were separated on 13% SDS/PAGE and transferred to polyvinylidenedifluoride (PVDF) membranes (Millipore, Burlington, MA, USA) for immunoblot analysis. Membranes were incubated with primary antibodies overnight, washed, and incubated with peroxidase-conjugated secondary antibodies at room temperature for 45 min. Reactive proteins were revealed using an enhanced chemiluminescent detection system (Clarity Western ECL Substrate, Bio-Rad, Hercules, CA, USA) and visualized on a Chemidoc Touch imaging system (Bio-Rad, Hercules, CA, USA). Densitometry analysis was performed using Image Lab (Bio-Rad, Hercules, CA, USA). Red Ponceau staining was used to verify total protein loading across the lanes and to normalize the protein content in each lane. AQP4ex, pAQP4ex, and AQP4 expression levels were represented as percentage changes from the baseline level, set at 100% [13,43].

### 4.9. Statistical Analysis

Mean ± standard error is reported in the results. Statistical analysis was performed using GraphPad Prism 9 (GraphPad, La Jolla, CA, USA) by Analysis of variance (ANOVA) followed by Tukey’s test. A *p* value < 0.05 was considered statistically significant.

## Data Availability

The original contributions presented in this study are included in the article. Further inquiries can be directed to the corresponding author.
